# Nonpyrogenic “Black” Nitrogen Likely Plays a Major Yet Underrecognized Role in the Global Nitrogen Cycle

**DOI:** 10.1021/acs.est.5c15974

**Published:** 2026-04-07

**Authors:** João Vitor dos Santos, Aleksandar I. Goranov, Kyle M. Lambert, Theodoro da Rosa Salles, Susan J. Carter, Ann Pearson, Patrick G. Hatcher

**Affiliations:** † Department of Chemistry and Biochemistry, 6042 Old Dominion University, Norfolk, Virginia 23529, United States; ‡ School of Technology, University of Campinas, Limeira, São Paulo 13484-332, Brazil; § Department of Earth and Planetary Sciences, 1812 Harvard University, Cambridge, Massachusetts 02138, United States

**Keywords:** biomass oxidation, Fenton chemistry, nitrogen incorporation, black nitrogen, condensed aromatic nitrogen

## Abstract

Condensed aromatic nitrogen (ConAN) is commonly found in soil and aquatic environments. ConAN is broadly viewed by the biogeochemistry community as a combustion-derived product leading to its frequent designation as pyrogenic “black” nitrogen. Here, we provide evidence that ConAN can also form through nonpyrogenic pathways. In an experiment simulating biomass oxidation, pine wood boards assembled with iron nails were exposed to 12 years of natural environmental conditions. Nitrogen concentrations increased from 0.2 to 1.2 wt % with increasing proximity to the nail. Ultrahigh resolution mass spectrometry showed enrichment of nitrogen-containing compounds within the lignin and condensed aromatic domains. X-ray spectroscopic characterization revealed the development of C–N bonds and formation of pyridinic and pyrrolic nitrogen moieties. We propose plausible nonpyrogenic formation mechanisms for ConAN and perform back-of-the-envelope calculations suggesting that biomass oxidation yields a nonpyrogenic ConAN flux to soils of 3–63 Tg-N yr^–1^. These findings demonstrate that ConAN can form under fire-free environmental conditions, highlighting nonpyrogenic ConAN as an underrecognized but potentially major component of the global nitrogen cycle. More broadly, this study reveals a previously ignored pathway for the long-term incorporation of nitrogen into refractory “stable” organic matter in soils and other environments.

## Graphical abstract

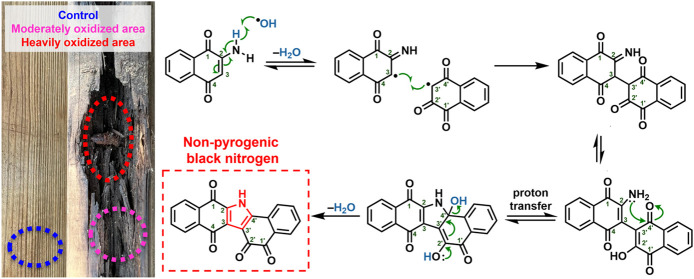

## 1 Introduction

The global pool of soil organic matter represents one of the largest reservoirs of nitrogen, a fundamental element for life and a central regulator of Earth’s biogeochemical cycles.^1–3^ Estimates of global soil nitrogen stocks in the upper 1 m are in the range of 95 – 140 Pg-N^1,4,5^ though values vary depending on quantification methods and assumptions, and uncertainties remain considerable. Because inorganic nitrogen typically represents only a minor fraction, the vast majority of soil nitrogen exists in various organic forms.^6^ To date, it remains unclear what mechanisms control the long-term retention and stability of nitrogen in the environment.

Among the diverse nitrogen functionalities present in soils, condensed aromatic nitrogen (ConAN) is likely the most stable form. ConAN refers to nitrogen incorporated into fused aromatic ring structures, typically occurring as heterocyclic nitrogen within polyaromatic frameworks in the forms of pyrrole and pyridine.^7–12^ Current estimates indicate that ConAN comprises 10 – 35% of soil organic nitrogen.^13,14^ Historically, ConAN has been viewed by the biogeochemistry community as a combustion-derived product originating from wildfires, fossil fuel combustion, and other types of biomass burning (e.g., prescribed fires). Evidence for this interpretation has been derived from multiple analytical techniques that identify nitrogen-bearing aromatic and heterocyclic structures in charred biomass and fire residues. This includes ^15^N solid-state nuclear magnetic resonance spectroscopy, nitrogen K-edge X-ray absorption spectroscopy, and photoelectron spectroscopy.^15–19^ Because of its assumed pyrogenic origin, ConAN is commonly referred to as “black” nitrogen (BN).^9^ Considering the stability of condensed aromatic species to microbial degradation,^20^ ConAN is regarded as an important long-term nitrogen sink in the environment.^15,16^ Its persistence is attributed to selective preservation processes^21,22^ allowing it to accumulate in soils and remain stable over millennial time scales.^23,24^ While the contributions to soil and aquatic reservoirs of condensed aromatic carbon (ConAC, the carbon analog of ConAN) from sources such as biomass burning^25^ and fossil fuels^26^ have been robustly quantified, no estimates exist for ConAN stocks or fluxes. Research to date has primarily addressed ConAC inventories,^27^ bulk soil total nitrogen stocks,^28^ or fire-related nitrogen dynamics.^9,29^

At the same time, the story about pyrogenic species appears to be more complex than previously thought. Even though ConAC is thought to be exclusively derived from combustion (i.e., being equivalent to pyrogenic “black” carbon, BC), numerous studies have reported that ConAC quantities in soils and rivers do not correlate with local fire histories.^30–34^ These discrepancies have suggested that additional, nonpyrogenic production processes exist that contribute to the environmental pool of ConAC, which can explain the global correlations between ConAC concentrations with soil organic carbon^33,35,36^ and fluvial dissolved organic carbon.^37,38^ About a decade ago, pioneering studies showed that ConAC can be produced abiotically during photochemical^39^ and dark Fenton oxidation of lignin.^40–42^ A more recent study showed quantitatively that such oxidation pathways in soil and fluvial environments are likely to produce considerable amounts of nonpyrogenic ConAC (e.g., ∼163 Tg-C yr^–1^ into soils).^43^ This suggests that the environmental reservoirs and fluxes of “true” pyrogenic ConAC are severely overestimated. Collectively, these previous findings demonstrate that the proposed nonpyrogenic pathways for condensed aromatic species formation are both chemically feasible and environmentally relevant, indicating that they are likely widespread and produce ConAC of substantial quantities.

In light of these findings, it is plausible that ConAN may also form nonpyrogenically under similar conditions.^9^ If both ConAC and ConAN can originate from environmentally ambient nonpyrogenic processes, their reliability as exclusive markers of combustion must be reconsidered, and their reservoirs and fluxes must be re-evaluated. Very few studies provide support for nonpyrogenic oxidative ConAN formation. In laboratory experiments, humic acids were reacted with ^15^N-labeled ammonium hydroxide and were shown to form pyrrolic nitrogen structures.^44^ Similar pyrrole moieties were observed in soil incubations with ^15^N-labeled nitrite.^45^ Additionally, heterocyclic nitrogen has been shown to form via Maillard-type reactions catalyzed by manganese oxides, particularly birnessite, at ambient temperatures.^46^ Biological sources of ConAN must be also acknowledged - small ConAN of 2–3 rings (azaarenes such as quinoline, benzo­[*h*]­quinoline, acridine, and carbazole) can be produced by rutaceous and nonrutaceous plants, fungi and other microbes, as well as animals,^47,48^ though larger ConAN are not known at present to have biological sources. While these studies clearly establish the feasibility of nonpyrogenic heterocyclic nitrogen formation though oxidative or biological pathways, their mechanistic controls, environmental relevance, and quantitative contributions to global ConAN pools remain poorly constrained.

A further complication is that heterocyclic nitrogen occurs in both condensed and noncondensed structures. For example, heterocyclic nitrogen occurs in DNA and RNA nucleotides, amino acids, porphyrin structures (e.g., in chlorophylls and cytochromes), and in other types of metabolites.^10–12,49^ These biogenic compounds only contain heterocyclic nitrogen structures of one or two rings (e.g., purines, pyrimidines, pyrroles) that do not constitute ConAN, which is found within structures of at least two fused aromatic rings that share a conjugated aromatic bond (e.g., ConAN is a quinoline-type structure at minimum). Purine, pyrimidine, or pyrrole moieties would be classified as ConAN if they exist within such condensed aromatic structures. Therefore, distinguishing small nitrogen-containing heterocycles from ConAN requires analytical techniques capable of differentiating aromatic structures of different sizes. This is a major challenge, and the previously applied ^15^N nuclear magnetic resonance and X-ray methods lacked the resolution needed to differentiate small aromatic compounds from ConAN structures.^11,49^ This limitation has contributed to the historical misinterpretation of biologically derived heterocyclic nitrogen as pyrogenic, which, in addition to not considering oxidation-derived ConAN formation, collectively necessitates a re-evaluation of the global nitrogen cycle.

In this study, we investigate nonpyrogenic ConAN formation in greater detail using advanced analytical techniques, including X-ray photoelectron spectroscopy (XPS), nitrogen K-edge X-ray absorption near edge structure (XANES) spectroscopy, solid-state nuclear magnetic resonance (NMR) spectroscopy, and Fourier transform–ion cyclotron resonance–mass spectrometry (FT-ICR-MS), with the latter technique being able to differentiate small aromatic compounds from ConAN structures. We explore the role of biomass oxidation in the formation of ConAN and hypothesize that oxidative transformation of lignin-rich biomass enhances its reactivity toward nitrogenous compounds, thereby promoting the abiotic formation of ConAN structures. Our results provide a mechanistic explanation for the formation of ConAN under nonpyrogenic environmental conditions indicating that nonpyrogenic sources likely contribute to environmental ConAN pools (i.e., ConAN is not necessarily equivalent to BN). We also present the first estimate for global nonpyrogenic ConAN fluxes to soils, which is an initial step toward the development of a global cycling framework for these previously overlooked nitrogen species.

## 2 Materials and Methods

### 2.1 Sample Collection

To investigate the nitrogenous compounds formed during long-term environmental oxidation, we examined wood boards from an outdoor deck structure in Norfolk, Virginia, USA. The deck, constructed from commercially purchased pine wood (exact species unknown) and assembled with iron nails, had been exposed for approximately 12 years to environmental conditions, including repeated wetting-drying cycles and temperature fluctuations. Thus, this wood-nail setup can be viewed as a model for studying biomass oxidation, as metallic iron is continuously corroded by oxygen and water, releasing reactive oxygen species such as hydroxyl radicals and superoxide.^50^ Although some iron likely leached over time, the nails provided a substantial iron reservoir and remained largely intact after 12 years (Figure 1). The variable pH of rainwater^51^ likely further promoted the corrosion of the Fe nail and the conversion of metallic Fe into Fe oxyhydroxide precipitates, which can continuously catalyze Fenton reactions and sustain the strong oxidative conditions. Furthermore, in such a reactive system, semiquinone-driven redox cycling^52^ also likely occurred, generating additional reactive intermediates as well as hydrogen peroxide, which further contributed to the sustained Fenton oxidative conditions over extended periods. Hydroxyl radicals, superoxide, and hydrogen peroxide are environmentally ubiquitous and represent key intermediates in biomass oxidation during photochemical, biological, and mineral-driven degradation processes in terrestrial and aquatic systems.^39,53,54^

**Figure 1:**
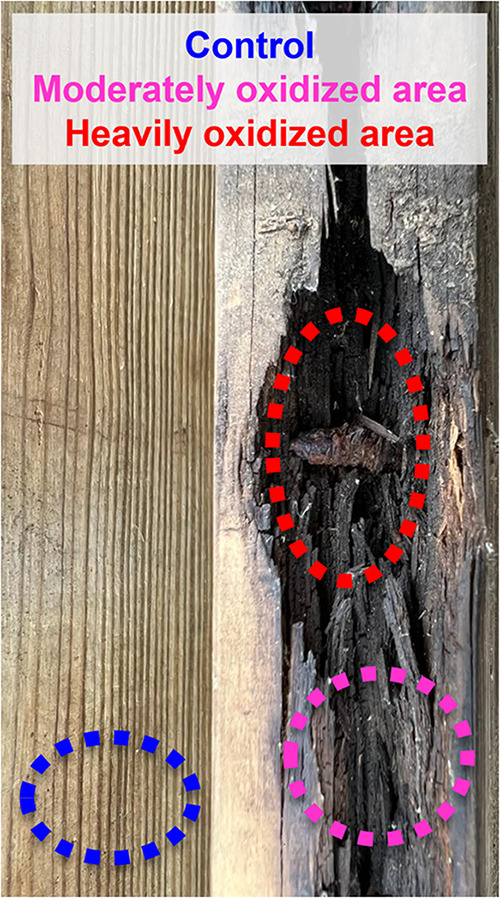
Wood-nail model system showing the formation of charcoal-like material after 12 years of environmental oxidation. Three samples representing an oxidation gradient were collected: pine wood control (blue), moderately oxidized wood (pink), and heavily oxidized wood (red).

When the deck structure was dismantled, the planks exhibited distinct blackened zones around areas in direct contact with the iron nails (Figure 1). These regions, reminiscent of charcoal, appeared to have undergone substantial oxidative transformation without the involvement of fire. The homeowner confirmed that the deck had never been exposed to fire or lightning strikes, thereby ruling out combustion or electricity-related thermal processes such as “Lichtenberg” burning. Growth of lichens, fungi, or other macroscopic organisms was not observed even though these wood boards had not been pressure treated. The nailed wood planks were located beneath a layer of boards, which limited sunlight exposure and thus minimized photochemical reactions. Further details about this wood-nail model system can be found in Goranov et al.,^43^ where it is referred as “Pine 1”.

To capture an oxidative gradient, we collected three samples based on color and proximity to iron nails. All samples were ground to fine powders prior to analyses.

- **Wood control** (**dashed blue circle**; Figure 1): A section with no visible discoloration, located far from any iron-induced alteration, and representing unoxidized, native pine wood material.
- **Moderately oxidized wood** (**dashed pink circle**; Figure 1): A section with slight to moderate darkening. It contains scattered black patches, but retains the features of the unaltered, original wood. This area was closer to the iron nail, indicating partial oxidative transformation.
- **Heavily oxidized wood** (**dashed red circle**; Figure 1): A section adjacent to an iron nail, with a distinctly black, charcoal-like appearance. Based on visual assessment, this appeared to have undergone a substantial “chemical charcoalification” indicative of a complete oxidative transformation.

### 2.2 Analytical Characterization

#### 2.2.1 Elemental, Molecular, and Isotopic Characterization

Elemental analysis of carbon (C%) and nitrogen (N%) was performed using a Thermo Finnigan FlashEA 1112 elemental analyzer, calibrated with nicotinamide (CE Elantech, Inc.).

Condensed aromatic carbon (ConAC) was quantified using the benzenepoly­(carboxylic acid) (BPCA) method.^55^ Dried and homogenized samples, no more than 5 mg carbon-equivalents,^56^ were weighed in 20 mL glass ampules. Concentrated nitric acid (2 mL, 65% HNO_3_, J.T. Baker, trace metal grade) was added and the ampules were allowed to sit for 15 min.^55^ Ampules were then flame-sealed and thermolyzed in a programmable oven for 9 h at 170 °C.^57^ After the digestion, the nitric acid was evaporated at 60 °C in a sand bath under a gentle stream of ultrapure N_2_ gas (Airgas, UHP300). The BPCA-containing residue was then dissolved in 2 mL of 0.6 M phosphoric acid and filtered using a 0.2 μm PTFE filter into an autosampler vial. Benzenehexa- (B6CA) and benzenepentacarboxylic (B5CA) acids were quantified chromatographically on an Agilent 1100 high-performance liquid chromatography system using a gradient of 0.6 M phosphoric acid (pH = 1) and a phosphate buffer (20 mM, pH = 6) on an Agilent Poroshell 120 Phenyl-Hexyl (4.6 mm × 150 mm, 2.7 μm) column following Wagner et al.^55^ The measured quantities of B6CA and B5CA were related to the initial concentration of ConAC in the samples using a conversion factor of 7.04.^58^

Stable nitrogen isotope composition (δ^15^N) was measured on a Thermo Scientific Flash EA instrument equipped with a thermal conductivity detector and a Delta V Plus isotope-ratio mass spectrometer (IRMS). δ^15^N measurements were peak-size-corrected and scale-corrected using laboratory and authentic reference standards (glutamic acid: δ^15^N = −5.9 ‰; USGS40: δ^15^N = −4.52 ‰; USGS41a: δ^15^N = +47.55 ‰; and tyrosine: δ^15^N = +4.7 ‰). All results are reported in delta notation (‰) relative to atmospheric N_2_.

### 2.3 Structural Characterization

Carbon and nitrogen speciation were determined using X-ray photoelectron spectroscopy (XPS). Analyses were performed on a Thermo Scientific K-Alpha instrument. Survey spectra were collected with a 400 μm spatial resolution, a pass energy of 50.0 eV, and an acquisition time of approximately 2 min and 23 s (acquiring a total of 15 scans). The instrument operated with an Al Kα X-ray source in standard-lens and CAE analyzer modes. Data were acquired with an energy step size of 0.100 eV across 191 steps. High-resolution carbon 1s and nitrogen 1s spectra were acquired under similar conditions and subsequently deconvoluted using the Thermo Avantage software (version 5.957).

Nitrogen speciation was determined also using K-edge X-ray absorption near-edge structure (XANES) spectroscopy at the IPÊ beamline (Inelastic scattering and photoelectron spectroscopy) of Sirius, the fourth-generation synchrotron source at the Brazilian Synchrotron Light Laboratory (*Laboratório Nacional de Luz Síncrotron*, LNLS).^59^ Finely ground samples were homogenized and mounted on the sample holder using carbon tape. XANES spectra were acquired at the XPS station in total fluorescence yield (TFY) mode, with an energy resolution of 0.2 eV, an acquisition time of 0.5 s per point, and a beam spot size of 20 μm × 6 μm. Ten scans were collected for each sample and subsequently averaged. Spectra were processed using the Athena software (Demeter 0.9.26).

Additional structural information was obtained using solid-state ^13^C nuclear magnetic resonance (NMR) spectroscopy. ^15^N spectra could not be acquired due to the low nitrogen concentrations and high iron concentrations. ^13^C spectra were obtained using the nearly quantitative multiple-pulse cross-polarization method^60^ on a 400 MHz Bruker ADVANCE II spectrometer equipped with a 4 mm HCN probe. Samples were packed in 4 mm zirconium rotors sealed with Kel-F caps and spun at 14 kHz. Spectra were acquired using 4000 scans with a 1-s relaxation delay. Glycine was used externally for chemical shift referencing (COO resonance at 176.20 ppm). Six chemical shift regions corresponding to key functional groups were integrated: alkyl-C (0–45 ppm), methoxy-C (45–60 ppm), O-alkyl-C (60–110 ppm), aryl-C (110–145 ppm), O-aryl-C (145–165 ppm), carboxyl-C (165–185 ppm), and carbonyl-C (185 – 220 ppm).

### 2.4 Molecular-Level Characterization

To investigate the molecular composition of the three wood samples, alkaline extractions were conducted under an N_2_ atmosphere using 0.1 M NaOH (ACS certified grade). Samples were shaken at 170 rpm in the dark at 25 °C for 24 h and then filtered through precombusted 0.7 μm glass fiber filters (GF/F, Whatman, 47 mm diameter) to isolate liquid extracts required for FT-ICR-MS analysis. Extracts were diluted to 50 mg-C L^–1^ and mixed 1:1 with methanol (Optima LC-MS grade) to create methanol:water solutions at 25 mg-C L^–1^.

Samples were infused at 120 μL/h into an Apollo II electrospray ionization source (operating in negative-ion mode) coupled to a 10-T Bruker Daltonics Apex Qe FT-ICR-MS. Instrument calibration was performed using poly­(ethylene glycol)^61^ and validated with the Suwannee River Fulvic Acid standard^62^ from the International Humic Substances Society. Samples were analyzed over a mass range of 200 to 800 Da, with 300 scans per sample. Procedural blanks confirmed no carryover contamination. Electrospray voltages and ion accumulation delay were optimized individually to ensure consistent ionization and comparable ion detection across samples.

Mass spectra were processed by selecting ions with signal-to-noise ratios ≥ 3. Mass lists were calibrated using naturally abundant fatty acids, dicarboxylic acids, and CH_2_ homologous series.^63^ Peaks corresponding to process blanks, salts, doubly charged ions, and ^13^C isotopologues were removed. Molecular formulas were assigned within defined elemental ranges (^12^C_5‑∞_, ^1^H_5–100_, ^16^O_1–30_, ^14^N_0–5_, ^32^S_0–4_, and ^31^P_0–2_). Ambiguous assignments were refined through homologous series considering groups such as CH_2_, H_2_, COO, CH_2_O, O_2_, H_2_O, NH_3_. All formula assignments were maintained within ±1 ppm mass accuracy. All data analysis was performed using the Toolbox for Environmental Research “TEnvR”^64^ in MATLAB (version 2022a).

The modified aromaticity index (AI_mod_) was calculated for each molecular formula to classify formulas according to aromaticity.^65^ Formulas with AI_mod_ ≥ 0.67 and at least 15 carbons were defined as ConAC (or ConAN, if nitrogen-containing).^9^ Formulas with AI_mod_ between 0.50 and 0.67 were considered to be aromatic with aliphatic side chains, those between 0 and 0.50 were classified as olefinic, and formulas with AI_mod_ = 0 were categorized as aliphatic. Molecular formulas were further grouped based on the presence of different heteroatoms into CHO, CHON, CHOS, and CHOP categories and also assigned to biochemical classes based on H/C and O/C ratios, and the presence of nitrogen: lignin-like (AI_mod_ < 0.67; H/C < 1.5; 0.1 ≤ O/C ≤ 0.67), tannin-like (AI_mod_ < 0.67; H/C < 1.5; O/C ≥ 0.67), sugar-like (H/C ≥ 1.5; O/C ≥ 0.55; *N* > 0), protein-like (H/C ≥ 1.5; O/C < 0.55; *N* > 0), and lipid-like (H/C ≥ 1.5; O/C < 0.67; *N* = 0) formulas.^66^

## 3 Results and Discussion

### 3.1 Biomass Oxidation Produces a Reactive Structural Backbone

Spectroscopic analysis using XPS (Figure 2a–c) and NMR (Figure 2d,e) provided complementary information, revealing clear shifts in carbon functional groups along the oxidation gradient of the three wood samples. The XPS carbon 1s spectrum of the wood control (Figure 2a) was dominated by C–O bonds, which are typically associated with alcohols and ethers. C–C/C-H bonds, associated with various aromatic and aliphatic moieties, were also found. The C–O, C–C, and C–H bonds likely originated from lignin and carbohydrates^67^ as these are the main two structural biopolymers in woody biomass. The C–O bonds increased from 53% in the control to 65% in the moderately oxidized sample and then decreased to 42% in the heavily oxidized sample. This nonmonotonic trend suggests that O-rich oxidation products are intermediates that degrade upon continued oxidation. This observation is consistent with our previously proposed radical-driven oxidation mechanism, where organic compounds are initially hydroxylated (formation of alcohols, C–OH groups), then converted to aldehydes and ketones (HC=O and C=O groups). While ketones tend to be stable, aldehydes can further oxidize to carboxyl groups (COOH), which may ultimately be cleaved as CO_2_.^68^ Alternatively, this observed pattern may reflect spatial heterogeneity of C–O functionalities, as XPS probes only the sample surface layer.

**Figure 2:**
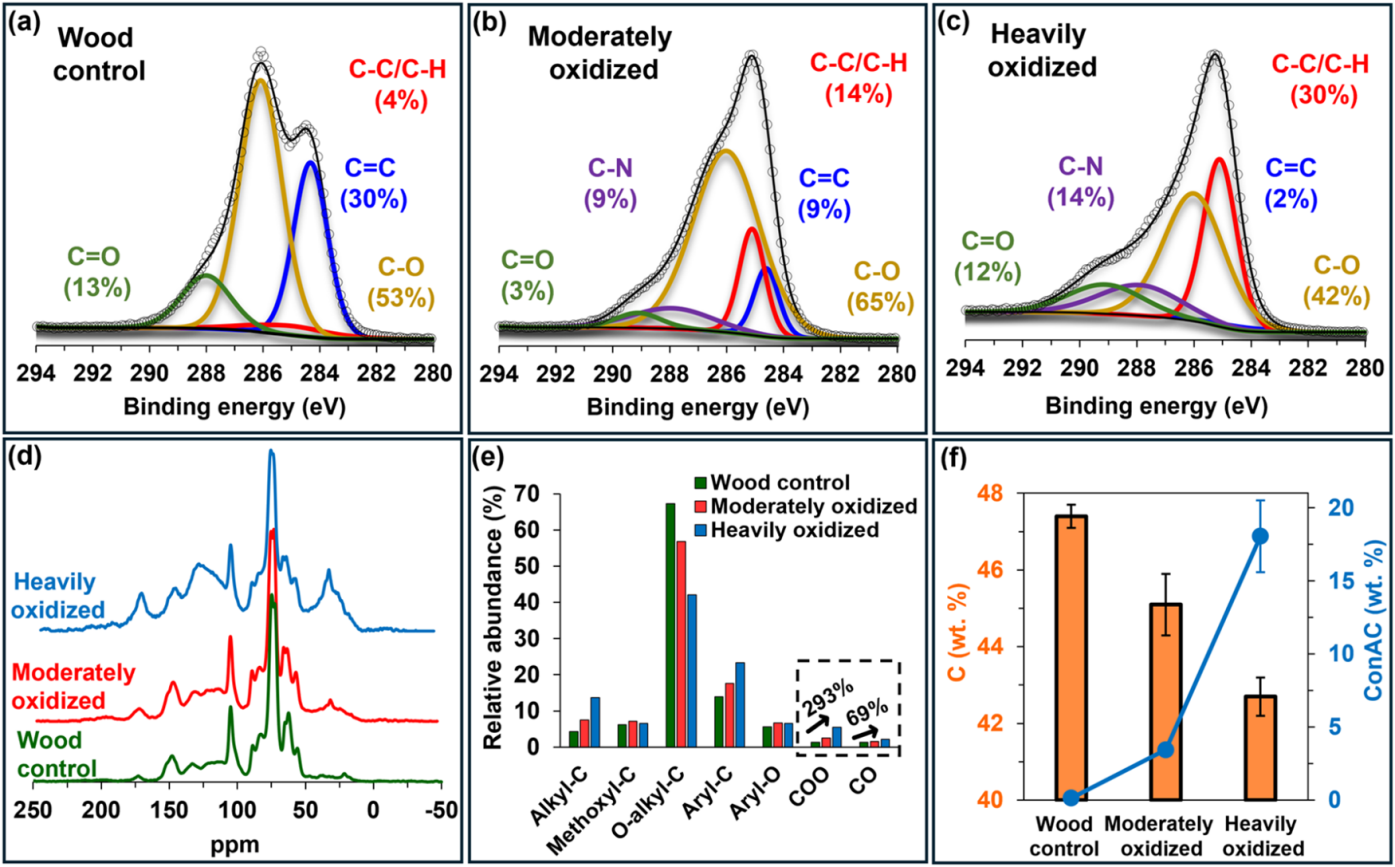
X-ray photoelectron spectroscopy (XPS), solid-state ^13^C nuclear magnetic resonance (NMR), and bulk quantitative analyses of the control, moderately oxidized, and heavily oxidized wood samples. Panels (a–c) shows the carbon 1s XPS spectra (whole spectra can be found on Figure S1 in the SI). Panel (d) displays the ^13^C NMR spectra, with panel (e) showing the relative abundance (%) of the seven functional regions derived from the NMR spectra. Panel (f) shows quantitative data on bulk carbon and condensed aromatic carbon (ConAC).

XPS analysis also revealed the emergence of C–N bonds during oxidation. These bonds were undetectable in the control sample (Figure 2a) but evolved to 9% in the moderately oxidized wood (Figure 2b) and 14% in the heavily oxidized wood (Figure 2c). This is a strong indicator that nitrogen was progressively incorporated into organic compounds with the increasing oxidation gradient.

Complementary structural information was obtained from solid-state ^13^C NMR (Figure 2d,e). The spectrum of the control wood showed high proportions of O-alkyl-C from carbohydrates and pronounced aryl-C resonances from lignin, confirming that unoxidized pine wood was rich in polysaccharide and lignin structures, consistent with previous studies on woody biomass.^69^ The persistence of these biopolymers after 12 years, particularly of the carbohydrates, provides key chemical evidence against microbial involvement in the transformations of organic matter in this wood-nail model system (Figure 1).

Oxidation led to clear spectral changes: O-alkyl-C from carbohydrates and lignin decreased while aliphatic alkyl-C (CH_3_, CH_2_, and CH groups) increased (Figure 2e). This is consistent with the expected features of biomass oxidation products.^68^ This increase in alkyl-C also correlates with the rise in C–C/C-H bonds observed by XPS (Figure 2a–c), suggesting that surface-sensitive XPS data are representative of the entirety of the sample. Additionally, resonances corresponding to COO (likely carboxylic acids) and CO (likely quinones) increased by 293% and 69%, respectively (Figure 2e). These functional groups are highly reactive and provide an organic backbone susceptible to covalent binding by nitrogen-containing nucleophiles.

Quantitative carbon analysis revealed a substantial loss of carbon from 47% in the control to 43% in the heavily oxidized sample (Figure 2f), as expected during biomass oxidation.^70^ enzenepoly­(carboxylic acid) (BPCA) analysis, a method specific for quantifying ConAC,^55^ showed an enrichment of up to 18 wt % in the most oxidized sample (Figure 2f). This indicates that condensed aromatic structures were produced during oxidation and is consistent with previous studies.^39,41,43^

### 3.2 Environmental Oxidation Promotes the Formation of Condensed Aromatic “Black” Nitrogen

Quantitative elemental and XPS analyses revealed progressive nitrogen enrichment with increasing oxidation (Figure 3a,b). Elemental analysis showed nitrogen increasing from 0.3% in the control to 1.2% in the heavily oxidized wood, leading to a sharp decrease in the C/N ratio from 202 to 42. The XPS data displayed a similar trend, though with different magnitudes, with nitrogen rising from 0.7% in the control to 2.5% in the heavily oxidized wood. The C/N ratio decreased from 96 to 28. These differences reflect the analytical windows of the two techniques as EA measures bulk composition while XPS probes the surfaces of sample grains. Despite this, both approaches consistently demonstrated a substantial nitrogen incorporation.

**Figure 3:**
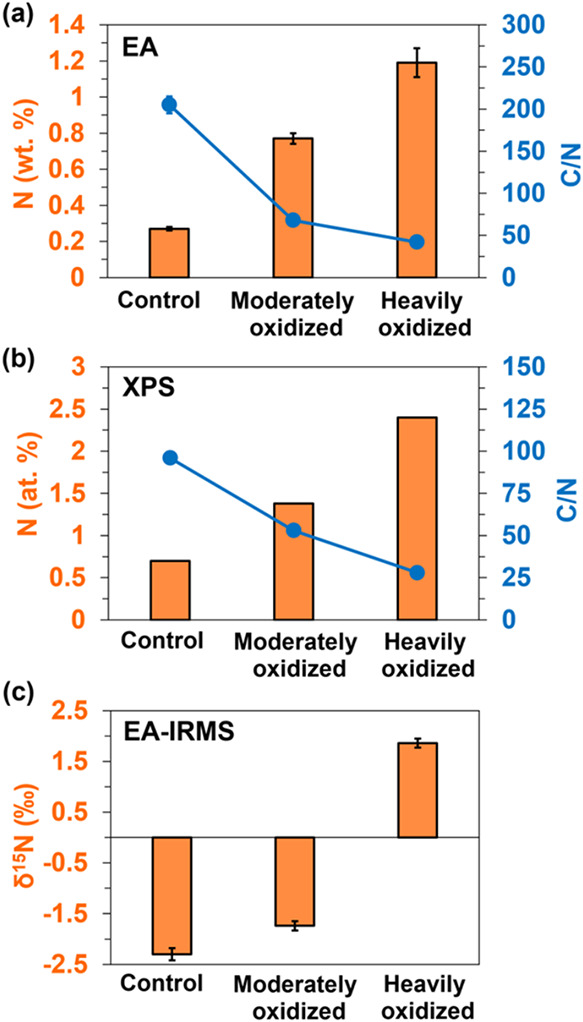
Nitrogen concentrations and C/N ratios determined by elemental analysis (a) and XPS (b). Panel (c) shows the stable nitrogen isotopic composition (δ^15^N) measured using elemental analysis coupled with isotope-ratio mass spectrometry.

These data support a framework in which oxidative transformations generate reactive domains, such as quinones (Figure 2e), that serve as sites for nitrogen immobilization through covalent bond formation.^71–73^ The immobilized nitrogen may originate from multiple sources, with atmospheric deposition via rainwater being the most likely contributor. Rainwater contains a complex mixture of nitrogenous compounds, including ammonia, nitrate, nitrite, and various organic species,^74–76^ which can all be incorporated into the oxidized woody biomass over the 12-year oxidation period.

Contributions from soil-derived nitrogen, microbial necromass, crop residues, or animal manure were unlikely^2^ as the deck was elevated above the soil surface. Although microbial activity is often linked to increasing nitrogen content and decreasing C/N ratios,^77^ microbial involvement in this system is negligible. Evidence for this is the lack of visible rotting (Figure 1) or altered chemical signatures of the control sample (Figure 2d) that would suggest the involvement of microbes. Furthermore, in a previous study^43^ the wood-nail model of this study was compared to another wood-nail model, the latter using pressure-treated pine wood (i.e., wood infused with a chemical that prevents microbial growth). The two wood-nail models exhibited virtually identical chemical trends. This concludes that microbes were not involved in the incorporation of nitrogen in the oxidized woody biomass observed in this study. This can be explained by the high iron concentrations in the system (higher than 4 wt %^43^), which generated high levels of reactive oxygen species that created unfavorable conditions for microbial survival.^78^ It must be noted, however, that there are microbes and plants that can produce small, ConAC and ConAN structures (e.g., naphthalene, perylene, azaarenes).^47,48,79–81^ Furthermore, microbes can secrete extracellular enzymes^82,83^ that produce reactive oxygen species^84,85^ and can perform biological oxidation, which can be another pathway for creating reactive backbones to which nitrogen can chemically bind. Thus, future work should investigate potential biological contributions of ConAN in oxic systems and assess whether ConAN, beyond small 2–3 ring azaarenes, can be produced biologically in the absence of oxidative pathways (e.g., through the use of radical quenchers).

Stable nitrogen isotopic composition (δ^15^N) increased along the oxidation gradient, from −2.50 ‰ in the control to −1.40 ‰ in moderately oxidized and to +1.60 ‰ in heavily oxidized wood (Figure 3c). This trend indicates the preferential loss of ^14^N-enriched species through leaching, volatilization, or abiotic transformations, leaving a residual pool enriched in ^15^N-bearing compounds.^74–76^ The δ^15^N of soils typically follows a similar trend along depth gradients, with progressive enrichment in ^15^N as organic matter is transformed and stabilized in deeper horizons.^74,75^ Such progressive isotopic ^15^N-enrichment points to a selective preservation mechanism, where nitrogen species resistant to degradation, such as ConAN, may accumulate in heavily oxidized zones. This is likely due to continuous oxidation producing oxidation-refractory ConAC and ConAN as previously shown during maple wood oxidation.^43^ Overall, this agrees with what is expected for condensed structures in soils based on their radiocarbon age on the order of millennia.^86,87^ Microbial involvement is ruled out as diazotrophs (nitrogen-fixing microbes converting atmospheric nitrogen into ammonia) fractionate the δ^15^N composition and the resultant ammonia has a more negative δ^15^N value (ranging −4 to −1 ‰^88^) relative to the δ^15^N of atmospheric nitrogen (0 ‰),^89^ a trend that is opposite to the one observed over the oxidation gradient in this study (Figure 3c).

As rainwater is suspected to be the main nitrogen source, modeling nitrogen incorporation using δ^15^N is challenging due to the wide spatial and temporal variability in rainwater δ^15^N values. For example, nitrogen in rainwater from an urban site in Guiyang, China ranged from −15.7 ‰ to +7.4 ‰, being more ^15^N-depleted during warmer months and more ^15^N-enriched during cooler months.^76^ Thus, it is not possible at present to set end-member values and model the isotopic changes. Furthermore, we have recently shown that stable carbon isotopic composition (δ^13^C) can be altered by oxidation caused by the preferential loss of ^12^C to CO_2_ or other gases.^70^ Thus, the changing isotopic pattern in δ^15^N observed in our experiment could also possibly reflects isotopic fractionation associated with oxidation and subsequent loss of ^14^N-enriched substances. Future N-incorporation studies should consider these pioneer trends and perform a detailed isotopic characterization of samples (rainwater, biomass substrate, etc.) for successfully developing a robust isotopic model.

Ultrahigh-resolution mass spectrometry (FT-ICR-MS) was used to determine the type of nitrogen-containing compounds in base-extracts of the three samples. Molecular fingerprint maps (van Krevelen diagrams, Figure 4) revealed that CHO species dominated all samples but decreased from 91% in the wood control to 84 – 85% in oxidized wood samples (Table S1). Importantly, this decline coincided with an increase in nitrogen-containing formulas (i.e., CHON), which rose from only 4% in the control to 11–13% in the oxidized samples (Table S1). CHOS and CHOP species remained minor (<4%) and showed no consistent trends (Table S1).

**Figure 4:**
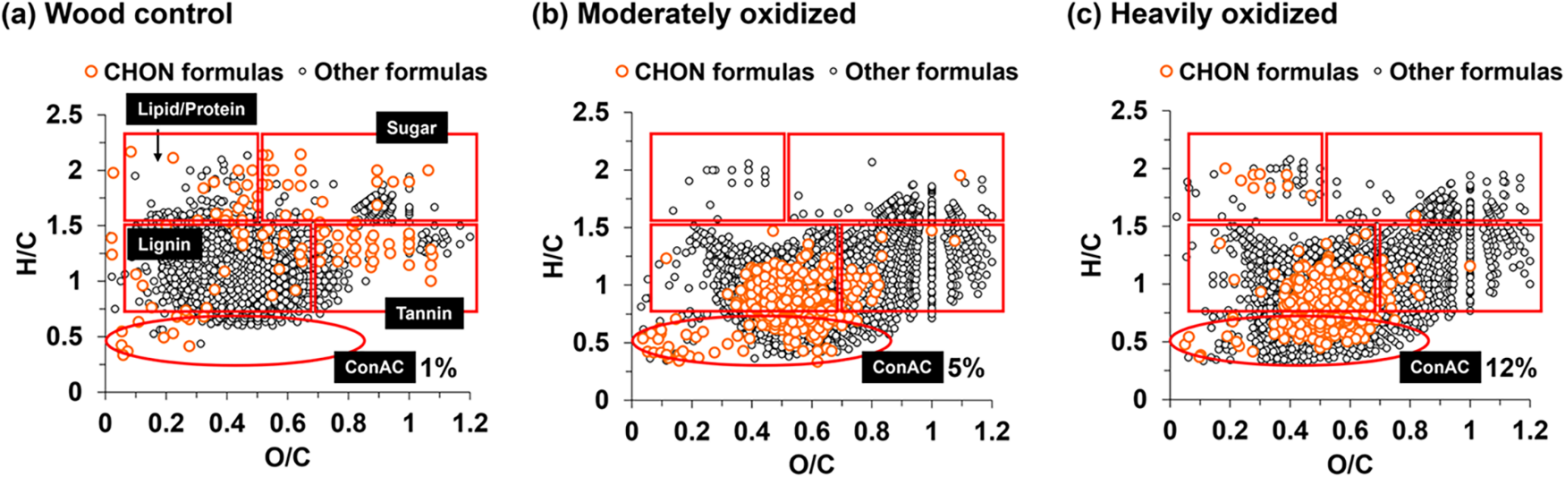
Van Krevelen diagrams showing molecular formulas identified in basic extracts of the control (a), the moderately oxidized (b), and the heavily oxidized wood (c) samples. The percentages next to the ConAC circles indicate the number of ConAC formulas relative to the total number of formulas. The distribution of formulas into all biochemical classes as well as differentiation into CHON or other formulas are listed in Tables S1–S2.

Across all samples, lignin-like formulas were the most abundant class but decreased from 82% in the control to 61 – 63% in oxidized wood samples (Table S1). Oxidation shifted lignin-like compounds toward higher O/C ratios (Figure 4b,c) indicating a progressive oxidative functionalization such as carboxyl-group formation.^41^ Tannin-like formulas, representing oxidized lignin products, increased substantially from 5% in control to 19 – 24% in the oxidized samples (Table S1). Although native carbohydrates ionize poorly, their oxidized derivatives were more readily detected in the oxidized wood samples (Figure 4b,c).

The largest compositional shift occurred during the early stages of oxidation, as observed between the control and moderately oxidized sample (Figure 4a → Figure 4b). The Van Krevelen diagrams for the moderately and heavily oxidized samples were broadly similar indicating that prolonged oxidation (Figure 4b → Figure 4c) produced comparatively smaller changes in the overall molecular distribution. The main distinction was the progressive increasing in ConAC formulas – from 1% in the control (Figure 4a) to 5% in the moderately oxidized sample and to 12% in the heavily oxidized wood (Figure 4b,c). This trend is consistent with the quantitative BPCA results (Figure 2f) and supports our previous findings that prolonged oxidation promotes the production of ConAC.^43^ ConAC formulas varied in their nitrogen and oxygen contents: the moderately oxidized sample contained more N_2–5_ formulas with relatively low number of oxygens, whereas the heavily oxidized sample contained a higher abundance of N_1_ formulas with higher oxygen counts (Figure S4c). These molecular changes indicate a progressive change in the ConAC class of compounds with increasing oxidation, which could potentially be a cleavage of labile nitrogen groups (e.g., amino) and formation of stable groups, such as pyrroles, at prolonged oxidation.

To identify the types of structures associated with covalently bound nitrogen, we specifically focused on assessing CHON formula distributions on the van Krevelen plots (shown in orange on Figure 4), with all other detected formulas shown in black. More specifically, CHON species were evaluated across six biochemical class categorizations: lignin, ConAC, tannin, lipid, sugar, and protein (Table S2). In the wood control, CHON formulas were sparse, widely dispersed, and showed no clustering in specific biopolymer regions, which is consistent with minimal and nonspecific nitrogen association in woody biomass.^68,90^ Following oxidation, lignin-like formulas dominated the CHON pool, accounting for 78 – 80% of all CHON species, while tannin-like formulas (i.e., oxidized lignin species) accounting for 7 – 9% of the CHON species (Table S2). Lignin and its oxygen-rich oxidation products are recognized as key drivers for nitrogen immobilization.^71^ Notably, CHON abundance also increased in the ConAC region, reaching 10 – 12% (Figure 4b,c and Table S2). Together with evidence from protein-quinone interactions,^71^ dissolved organic matter in urea-fertilized pasturelands,^91^ and studies on polycyclic aromatic hydrocarbons interacting with nitrogenous compounds,^9,72,92–94^ our findings indicate that oxidized lignin and ConAC play a central role in nitrogen immobilization. These results show that the newly produced CHON compounds were aromatic, either lignin-like or condensed aromatic (i.e., as ConAN).

Analogously to the commonly used H/C and O/C ratios for generating van Krevelen diagrams,^66^ H/N and O/N ratios can be used to further highlight oxidation and unsaturation degrees of CHON species (Figure S2). Oxidized woods shifted toward more oxygenated, nitrogen-rich molecules with O/N ratios greater than 10 (Figure S2b,c). Most CHON species contained a single nitrogen atom (N_1_, Figure S3a), accounting for about 90% of CHON formulas in all samples. They were also heavily oxidized (O > 10), with a greater abundance of O_8_–O_15_ compounds within N_1_ formulas (Figure S3a). In lignin-rich regions (Figure S3b), highly oxygenated N_1_ species with oxygen counts from 8 to 15 (N_1_O_8_–N_1_O_15_) were especially abundant and displayed a Gaussian-like distribution (Figure S3b). This suggests a relatively uniform incorporation of nitrogen into oxygenated functionalities (e.g., quinones) of lignin. By contrast, N_1_ species in the ConAC region (Figure S3c) spanned a broader oxygenation range from 2 to 15 (N_1_O_2_–N_1_O_15_) and showed more variable abundances, consistent with a more heterogeneous nitrogen incorporation pathway into ConAC structures.

While FT-ICR-MS identified the molecular classes most enriched in nitrogen, it does not provide information on the specific bonding environments of nitrogen atoms (i.e., it cannot distinguish amides, amines, heterocyclic, or other types of nitrogen). To gain compositional insight into the nitrogen speciation, we combined XPS and XANES analyses (Figure 5). Nitrogen 1s XPS data could not be obtained for the wood control sample due its extremely low nitrogen content, which resulted in a poor signal-to-noise (Figure 5a). This is expected as woody biomass is particularly poor in nitrogen.^95^ By contrast, XPS spectra of the oxidized wood samples were of much higher quality enabling peak deconvolution and providing a clear picture of the nitrogen speciation (Figure 5b,c). In the moderately oxidized wood, three distinct nitrogen species were resolved: pyridinic nitrogen at 398.7 eV (12%), amine nitrogen at 399.9 eV (12%), and pyrrolic nitrogen at 400.7 eV (76%). The heavily oxidized wood spectrum was dominated exclusively by pyrrolic nitrogen indicating that progressive oxidation strongly favors pyrrolic nitrogen formation. This reduction in diversity of nitrogen types parallels the observed progression from N_2–5_ formulas to N_1_ with increasing oxidation, especially for the ConAC domain (Figure S3c).

**Figure 5:**
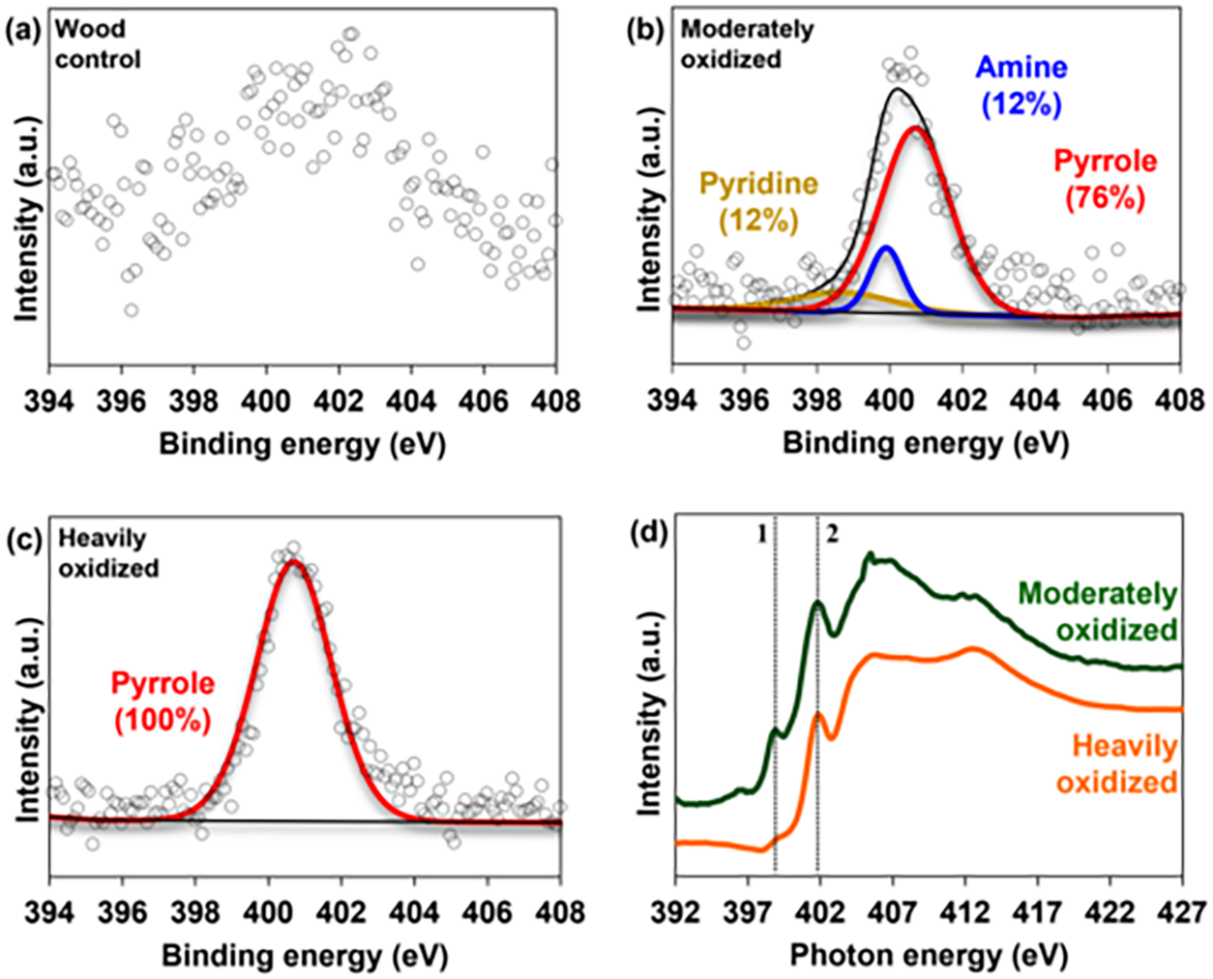
High-resolution nitrogen 1s XPS spectra of the (a) control wood, (b) moderately oxidized wood, and (c) heavily oxidized wood. Panel (d) shows the nitrogen XANES spectra of the oxidized samples (d), with 1 = pyridinic nitrogen, 2 = pyrrolic nitrogen.

Nitrogen XANES measurements further supported the XPS observations and provided complementary information (Figure 5d). Due to the low concentration of nitrogen in the control, only the oxidized samples were analyzed. The moderately oxidized wood displayed two prominent π* resonances at 399 and 402 eV. The lower-energy peak is consistent with pyridinic nitrogen (labeled as “1” on Figure 5d) whereas the higher-energy peak corresponds to pyrrolic nitrogen^96^ (labeled as “2” on Figure 5d). In the heavily oxidized wood, only the higher energy π* resonance at 402 eV was observed. This indicates that the nitrogen in the highly oxidized sample was primarily of pyrrolic form, which agrees with the XPS results (Figure 5c).

### 3.3 Proposed Mechanistic Pathways of Nonpyrogenic ConAN Formation

Our data shows that nitrogen can be immobilized within lignin derivatives (CHON formulas in the lignin region; Figure 4b,c) or within ConAC (CHON formulas in the ConAC region; Figure 4b,c). We propose that ConAN originates primarily from the initial incorporation of nitrogen into oxidized lignin and ConAC species, followed by subsequent reactions that generate pyrrole moieties (i.e., ConAN). There are several mechanistic possibilities for this and herein we suggest two plausible pathways for these processes using ammonia as an example nucleophile. These pathways, (one being radical-based and the other being two-electron-based) involve the formation of C–N bonds (matching carbon 1s XPS data, Figure 2b,c) followed by other chemical reactions that produce stable ConAN species such as pyrroles (matching nitrogen 1s XPS data, Figure 5b,c). A plausible route to nitrogen incorporation into quinones resultant from lignin oxidation is depicted in Figure 6. Schiff bases or enamine tautomers can be formed by reacting 2-hydroxy-1,4-napthoquinone structural units with ammonia or other nucleophiles (incorporation pathway A).^97^ Another alternative is to undergo Michael addition of ammonia or other nucleophiles into 1,4-napthoquinone structural units (incorporation pathway B).^98^ Further condensation into pyrroloquinones^99^ is likely to proceed by both radical (Figure 6, condensation pathway I) and two-electron pathways^100^ such as the well-established Knorr pyrrole synthesis^101^ (Figure 6, condensation pathway II).

**Figure 6:**
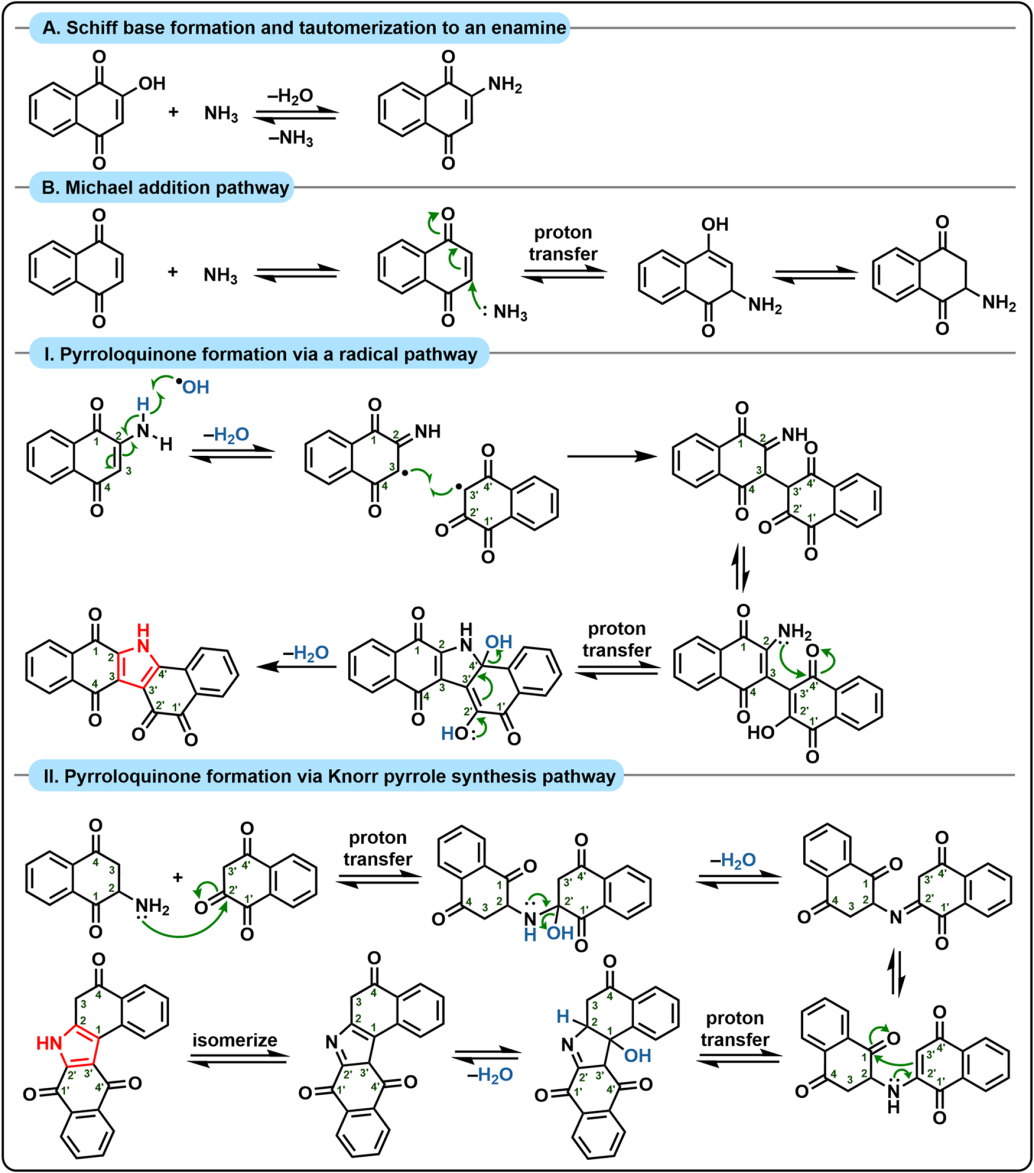
Mechanisms by which nitrogen is immobilized into quinones in oxidized lignin followed by formation of condensed aromatic nitrogen (ConAN) via the formation of pyrrole-containing structures (pyrrolic units colored in red).

Under the same oxidative conditions that promote quinone formation from lignin or ConAC, these systems are also likely to interact with initially formed enamines. Reactive oxygen species, such as hydroxyl radicals, can abstract hydrogen atoms, generating both *N*-centered and *C*-centered radicals. These radicals may subsequently undergo biaryl coupling, positioning the nitrogen atom in close proximity to an electrophilic carbonyl carbon (five atoms away in pyrrole-derived systems and six in pyridine-derived systems). This arrangement favors intramolecular condensation with the loss of water, ultimately yielding pyrroloquinone structures (Figure 6, I).

Alternatively, Michael adducts such as 2-aminoketones, formed from the reaction between quinones and ammonia (or other nitrogen-containing nucleophiles), may further react with additional quinone equivalents to generate Schiff bases. When derived from cyclic ketones, these intermediates readily tautomerize to enamines, which act as effective nucleophiles in condensation reactions with ketones. This process can ultimately lead to the formation of pyrroloquinones through intramolecular condensation with carbonyl groups via a classic Knorr-type pyrrole synthesis, accompanied by the loss of water.

These proposed mechanistic pathways for nonpyrogenic ConAN formation closely reflect the chemical environment of the oxidized wood samples investigated in this study. While we use ammonia as an example, these suggested mechanisms can operate with various forms of nitrogen naturally present in soils, such as ammonia, ammonium, nitrate, nitrite, amino acids, proteins, and others. Any of these species can act as a base and be a nucleophile. Mildly acidic conditions (pH = 4 – 6), typical of many soils, will favor the formation of protonated species (e.g., ammonium over ammonia). These conditions can catalyze the formation of Schiff bases,^102^ and, through Le Châtelier’s principle during reactions with carbonyl groups, promote the continuous regeneration of the unprotonated species (e.g., ammonia). Thus, even at low concentrations, this dynamic equilibrium could have provided a sustained supply of reactive ammonia or other nitrogen-containing compounds over extended periods. In the 12 years of environmental exposure, such flux likely supported slow, ongoing reactions between nitrogen-containing nucleophiles with oxidized lignin and ConAC explaining the observed stable nitrogen immobilization under natural conditions.

Although the actual compounds and reaction networks occurring in natural systems are undoubtedly more complex, the principles suggested here are supported by our data set and provide a conceptual framework for understanding how nitrogen may become immobilized and ultimately converted into ConAN (analogous to BN) under prolonged oxidative conditions. Future studies employing model compounds, isotopic labeling, and advanced structural identification techniques (e.g., quadrupole – time-of-flight analyses) should further elucidate the precise mechanisms underlying ConAN formation in the environment.

### 3.4 Back-of-the-Envelope Estimates for ConAN Fluxes to Soils

While ConAC can be quantified using established techniques such as the BPCA method,^55^ no equivalent method exists for ConAN. As a result, the contributions of ConAN to the global nitrogen cycle (i.e., the ConAC fluxes) remain poorly constrained. Therefore, at present global ConAN fluxes can be only approximated using indirect back-of-the-envelope calculations. Here, we combine published data with our novel data set to produce the first estimates for ConAN fluxes to soils.

Assuming that all soil ConAN is of pyrogenic origin, we combined the global flux of ConAC to soils (128 ± 84 Tg-ConAC yr^–1^)^38^ with the C/N ratio of condensed structures in soils (9–23)^103^ to estimate a global pyrogenic flux of ConAN of 2–17 Tg-ConAN yr^–1^ (Table S3). Following the common terminology in the wildfire literature, this flux would correspond to BN.

Using the multi-instrumental data set of this study, we build on this framework by providing a first estimate for the nonpyrogenic ConAN flux to soils (Table S4). We first estimate the global nonpyrogenic ConAC flux to soils. To do so, we combine the global biomass inputs to soils (44 Pg-C yr^–1^ of plant litter inputs^104^ + 25 Pg-C yr^–1^ of root exudates^105^ = 69 Pg-C yr^–1^) with the nonpyrogenic ConAC formation rates from this and our previous study^43^ (BPCA concentration divided by years of oxidative exposure = 0.063–1.505% ConAC yr^–1^; Table S4). We then combine the resulting annual nonpyrogenic ConAC flux (44–1043 Tg-ConAC yr^–1^) with the C/N ratio of nonpyrogenic ConAN structures identified using FT-ICR-MS (average of 19; Figure 4) to calculate a nonpyrogenic ConAN flux of 3–63 Tg-ConAN yr^–1^. This flux is comparable to the pyrogenic ConAN (BN) flux of 2–17 Tg-ConAN yr^–1^. Considering that the estimated ConAN flux may reach up to 63 Tg-ConAN yr^–1^, it is possible that soil ConAN is largely of nonpyrogenic origin, as previously proposed for ConAC.^43^ For this reason, we advocate against the use of terminologies such as “black carbon” and “black nitrogen” for ConAC and ConAN, respectively, as these structures could largely originate from nonpyrogenic processes.

Our estimates should be regarded as preliminary approximations due to several caveats and assumptions. Quantification of ConAN remains indirect, because no dedicated methodology currently exists, and future research should focus on developing approaches similar to the BPCA method. The scarcity of ConAC data and, in particular, ConAN data, limits the exploration of spatial and compositional variability in environmental samples. Furthermore, the data set of nonpyrogenic ConAC production is limited to five samples undergoing semicontrolled Fenton oxidation, which does not account for other processes such as photochemical or biological oxidation. Consequently, our estimates are meant to illustrate potential magnitudes rather than precise fluxes, and future studies should build upon these calculations to obtain robust measurements of ConAC and ConAN fluxes, stocks, and turnover rates. The main message is conceptual: the nonpyrogenic formation of ConAN appears to be a major process in soils and other environments. This process is currently overlooked in the global biogeochemical cycles as an important sink for carbon and nitrogen, particularly because condensed structures are highly refractory to degradation^20,106,107^ and exhibit very old radiocarbon ages, on the order of millennia.^86,87^

### 3.5 Environmental Implications

The multifaceted analytical approach used in this study demonstrates that increasing oxidation leads to a marked accumulation of ConAC and ConAN. Because they are generally considered to be produced exclusively through combustion, they are usually equated to BC and BN, respectively. By definition, BC and BN are formed during combustion processes.^16,108,109^ However, ConAC and ConAN have also been reported to form nonpyrogenically through oxidation^9,41,43,45,46,110^ and our study further explores this from a mechanistic and quantitative perspective. The “non-pyrogenic charcoalification” that we observe (Figure 1) indicates that ConAC and ConAN cannot be viewed as equivalents to BC and BN, respectively, and that their measurements cannot be directly used in modeling of wildfire-derived stocks and fluxes.

Furthermore, stable isotope analysis revealed a δ^15^N pattern resembling what is typically observed during biomass combustion.^111–113^ In fire-affected systems, isotopic fractionation occurs, because nitrogen-containing compounds are volatilized in ash and smoke, which preferentially releases ^14^N, leaving charred residues enriched in ^15^N.^111,112^ Consequently, postfire soils exhibit elevated, often positive δ^15^N values, and plants growing in these soils inherit this enriched isotopic signal.^113^ In our study, however, the same isotopic enrichment emerged without combustion and intensified with increasing oxidation. This shows that natural oxidative processes alone can drive ^14^N losses and ^15^N enrichment, which creates a new challenge for the interpretation of δ^15^N signatures in soil organic matter in the context of fire histories.

Using our novel data, we provide a first approximation of nonpyrogenic ConAN fluxes to soils, which has not been previously attempted due to the absence of direct quantification methods. We estimate a nonpyrogenic ConAN flux of 3–63 Tg-N yr^–1^, substantially higher than the estimated pyrogenic ConAN (i.e., BN) flux of 2–17 Tg-N yr^–1^. Despite substantial uncertainties, including indirect quantification, extrapolation from model systems, and variability in global biomass inputs, these estimates suggest that nonpyrogenic ConAN may represent a previously overlooked but potentially major nitrogen sink in soils. This suggests that there is a continuous sink of nitrogen in the global nitrogen cycle, which provides a complementary and persistent source of stable soil nitrogen that operates independently of fire events.

A key unresolved question concerns the bioavailability of nitrogen bound in ConAN structures formed through nonpyrogenic pathways. Notably, ConAC forms early during the oxidation (Figure 4b) and persists through the prolonged and more severe oxidative conditions (Figure 4c). Understanding the long-term stability of condensed structures and both their ConAC and ConAN pools will be critical for refining nitrogen cycling models in both fire and nonfire affected soils. Our current data indicate that microbes are likely not involved in the production of nonpyrogenic ConAC and ConAN, although they may participate in their degradation once formed. Future work should aim to reproduce these transformations under controlled biologically active conditions to assess the formation and persistence of nonpyrogenic ConAN.

## Supplementary Material



## Notes

Survey XPS spectra, categorization of molecular formulas from FT-ICR-MS, additional figures visualizing FT-ICR-MS data, tables for the calculation of ConAN fluxes (PDF)
