# Supplementary material for: Nonpyrogenic “Black” Nitrogen Likely Plays a Major Yet Underrecognized Role in the Global Nitrogen Cycle
Source: Environ Sci Technol. 2026 Apr 7;60(15):11486–99. doi: 10.1021/acs.est.5c15974 (PMC13104175; doi:10.1021/acs.est.5c15974)
Supplement: Supplementary file 1 [file es5c15974_si_001.pdf]

Non-pyrogenic “black” nitrogen likely plays a major yet underrecognized role in the global nitrogen cycle

João Vitor dos Santos[a*], Aleksandar I. Goranov[a], Kyle M. Lambert[a],
Theodoro da Rosa Salles[b], Susan J. Carter[c], Ann Pearson[c], Patrick G. Hatcher[a*]

[a]Department of Chemistry and Biochemistry, Old Dominion University, Norfolk, Virginia 23529, United States
[b]School of Technology, University of Campinas, Limeira, São Paulo 13484-332, Brazil
[c]Department of Earth and Planetary Sciences, Harvard University, Cambridge, Massachusetts 02138, United States

Corresponding authors (*): João Vitor dos Santos (j1dossan@odu.edu) and Patrick G. Hatcher (phatcher@odu.edu)

Supporting Information (SI)

Summary: 6 pages, 3 figures, 4 tables.

Table of Contents

Section 1. X-ray photoelectron spectroscopy (XPS): Figure S1 .... S2

Section 2. Ultrahigh resolution mass spectrometry (FT-ICR-MS): Figures S2-S3, Tables S1-S2 .... S3

Section 3. Estimating pyrogenic and non-pyrogenic ConAN fluxes to soils: Tables S3-S4 .... S5

References S6

Section 1. X-ray photoelectron spectroscopy (XPS)

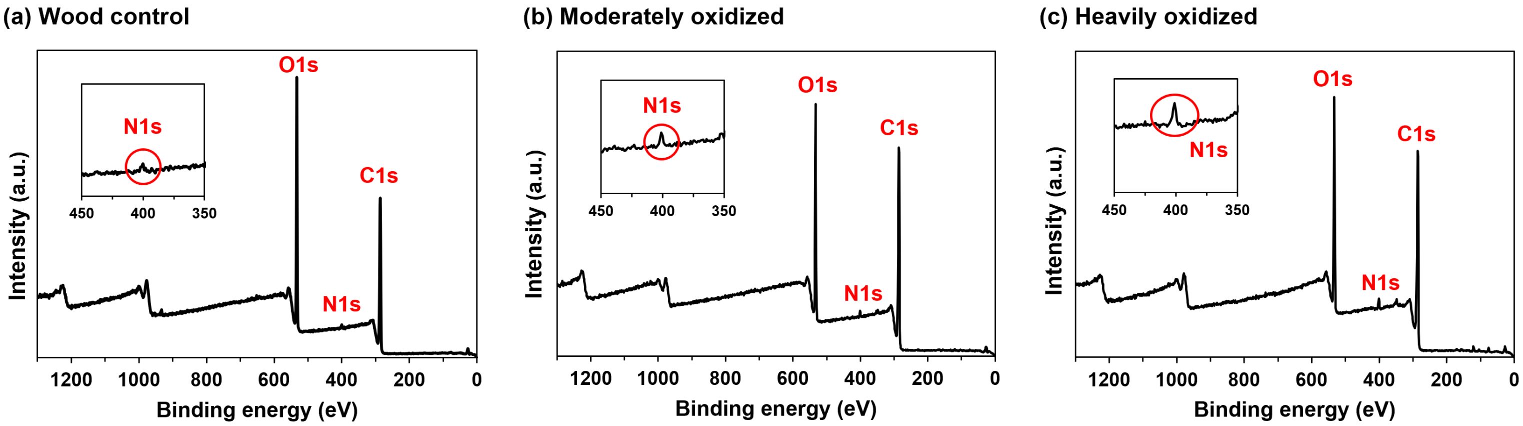

Fig. S1. X-ray photoelectron survey spectra of pine wood samples: (a) control, (b) moderately oxidized, and (c) heavily oxidized.

Section 2. Ultrahigh resolution mass spectrometry (FT-ICR-MS)

Table S1. Formulas in molecular and biochemical classes for wood control and oxidized samples.

		Wood control	Moderately oxidized	Heavily oxidized
**Molecular formula classes**	**# CHO**	2428 (91%)	2361 (84%)	2364 (85%)
	**# CHON**	109 (4%)	376 (13%)	308 (11%)
	**# CHOP**	62 (2%)	14 (1%)	14 (1%)
	**# CHOS**	85 (3%)	44 (2%)	68 (3%)
**Biochemical classes**	**# Lignin**	2212 (82%)	1769 (63%)	1686 (61%)
	**# ConAC**	19 (1%)	136 (5%)	305 (12%)
	**# Tannin**	120 (5%)	669 (24%)	525 (19%)
	**# Lipid**	192 (7%)	17 (1%)	29 (1%)
	**# Sugar**	102 (4%)	194 (7%)	189 (7%)
	**# Protein**	26 (1%)	0 (0)	9 (0)
	**# Other**	13 (0)	10 (0)	11 (0)

Table S2. Biochemical classes of only CHON molecular formulas for wood control and oxidized samples.

		Wood control	Moderately oxidized	Heavily oxidized
**CHON molecular formulas**	**# Lignin**	26 (24%)	301 (80%)	241 (78%)
	**# ConAC**	8 (7%)	38 (10%)	36 (12%)
	**# Tannin**	29 (27%)	35 (9%)	21 (7%)
	**# Lipid**	0 (0)	0 (0)	0 (0)
	**# Sugar**	17 (16%)	1 (0)	2 (1%)
	**# Protein**	26 (24%)	0 (0)	8 (3%)
	**# Other**	3 (3%)	1 (0)	0 (0)

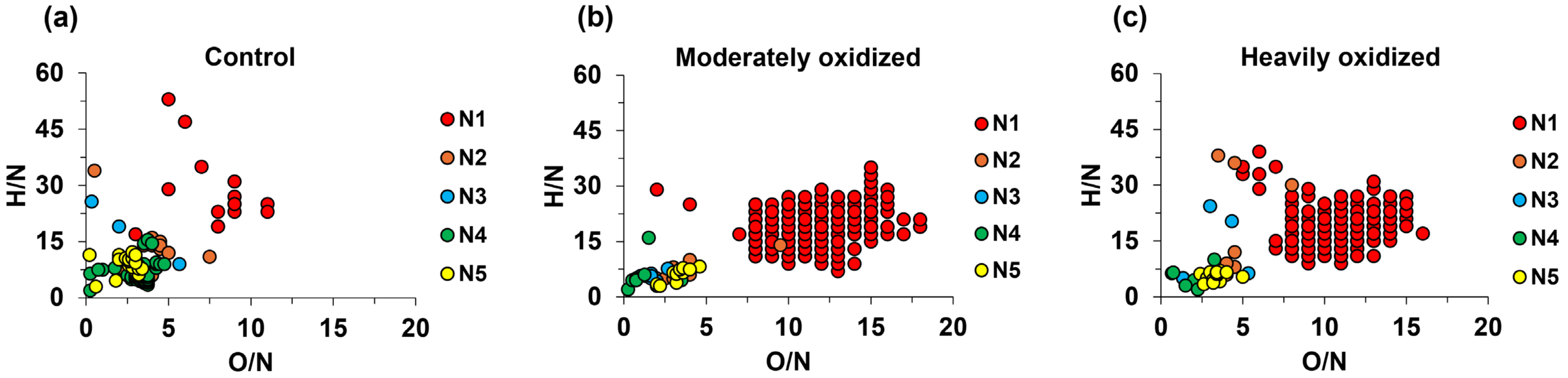

Fig. S2 H/N vs. O/N ratio plots (van Krevelen analogs for N) of nitrogen-containing formulas ($CHON_x$, where x = 1-5) in the (a) pine wood control, (b) moderately oxidized, and (c) heavily oxidized wood.

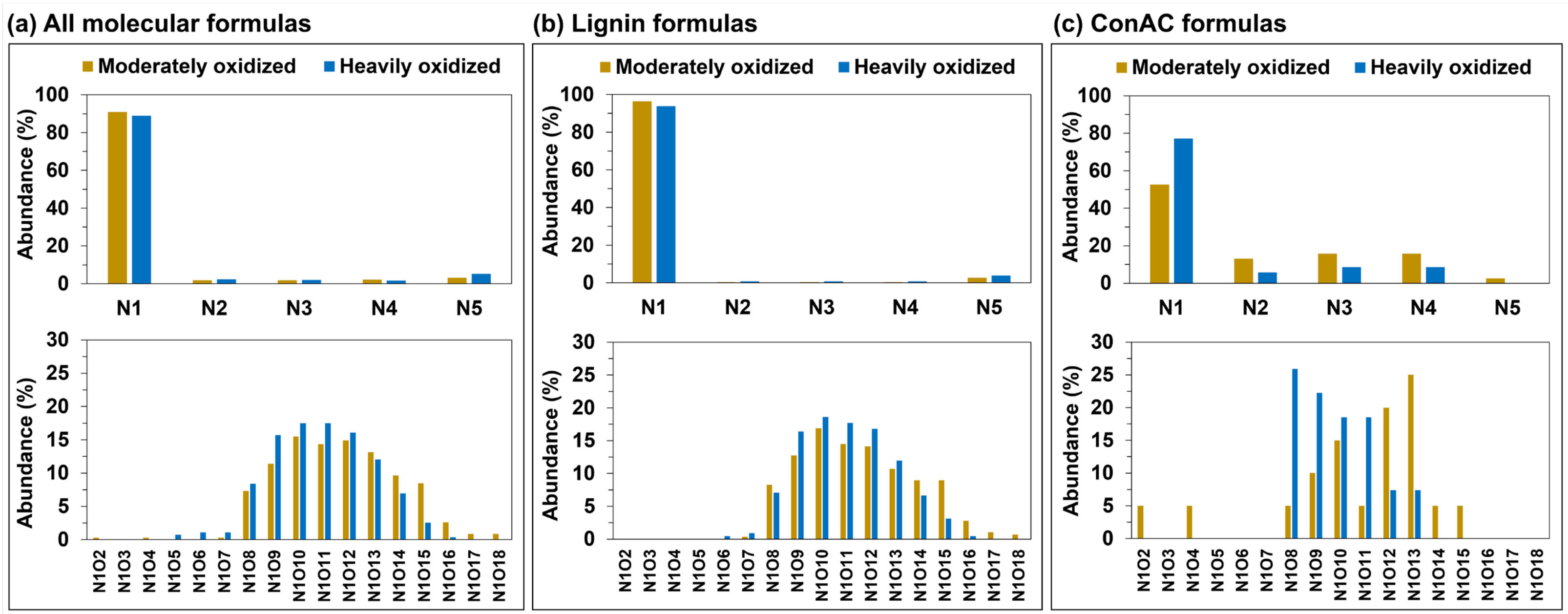

Fig. S3 Distribution of $CHON_x$ (top) and CHO_xN_1 classes (bottom) in (a) all formulas, (b) lignin formulas, and (c) ConAC formulas.

Section 3. Estimating pyrogenic and non-pyrogenic ConAN fluxes to soils

Table S3. Estimate of pyrogenic condensed aromatic nitrogen (ConAN) flux in soils (i.e., black nitrogen, BN).

Parameter	Value	Source
ConAC flux to soils	128 ± 84 Tg-ConAC yr^{-1}	Jones et al. [1]
C/N ratio of ConAC	16 ± 7	Determined by elemental analysis of chemothermal oxidation residues, Wang et al. [2]
ConAN flux	2 – 17 Tg-ConAN yr^{-1}	-

Table S4. Estimates of non-pyrogenic condensed aromatic nitrogen (ConAN) flux in soils.

	ConAC formation rate (% yr^{-1})	ConAC flux (Tg yr^{-1})	ConAN flux (Tg yr^{-1})	Source
Moderately oxidized	0.288	200	12	This study
Heavily oxidized	1.505	1043	63	This study
Pine 1	0.203	141	9	Goranov et al. [3]
Maple maximum	0.217	150	9	Goranov et al. [3]
Maple refractory	0.063	44	3	Goranov et al. [3]
Summary (range)	0.063 – 1.505	44 – 1043	3 – 63	-

Assumptions: Global plant litter + root carbon inputs = 69 Pg-C yr^{-1} (references [4,5]); average C/N ratio = 19.13 (based on FT-ICR-MS analysis of N-containing ConAC formulas in this study).

References

(1) Jones, M. W.; Coppola, A. I.; Santín, C.; Dittmar, T.; Jaffé, R.; Doerr, S. H.; Quine, T. A. Fires Prime Terrestrial Organic Carbon for Riverine Export to the Global Oceans. *Nat. Commun.* **2020**, *11* (1), 2791. https://doi.org/10.1038/s41467-020-16576-z.

(2) Wang, Q.; Liu, M.; Yu, Y.; Du, F.; Wang, X. Black Carbon in Soils from Different Land Use Areas of Shanghai, China: Level, Sources and Relationship with Polycyclic Aromatic Hydrocarbons. *Applied Geochemistry* **2014**, *47*, 36–43. https://doi.org/10.1016/j.apgeochem.2014.04.011.

(3) Goranov, A. I.; Chen, H.; Duan, J.; Myneni, S. C. B.; Hatcher, P. G. Potentially Massive and Global Non-Pyrogenic Production of Condensed "Black" Carbon through Biomass Oxidation. *Environ. Sci. Technol.* **2024**, *58* (6), 2750–2761. https://doi.org/10.1021/acs.est.3c05448.

(4) Gherardi, L. A.; Sala, O. E. Global Patterns and Climatic Controls of Belowground Net Carbon Fixation. *Proceedings of the National Academy of Sciences* **2020**, *117* (33), 20038–20043. https://doi.org/10.1073/pnas.2006715117.

(5) He, Y.; Wang, X.; Wang, K.; Tang, S.; Xu, H.; Chen, A.; Ciais, P.; Li, X.; Peñuelas, J.; Piao, S. Data-driven Estimates of Global Litter Production Imply Slower Vegetation Carbon Turnover. *Glob. Chang. Biol.* **2021**, *27* (8), 1678–1688. https://doi.org/10.1111/gcb.15515.
